# Modeling the formation of defensive gaps in basketball: Cutting on a teammate’s drive

**DOI:** 10.1371/journal.pone.0281467

**Published:** 2023-02-07

**Authors:** Bence Supola, Thomas Hoch, Arnold Baca

**Affiliations:** 1 Department of Sport Science, University of Vienna, Vienna, Austria; 2 Software Competence Center Hagenberg GmbH, Hagenberg, Austria; 3 Centre for Sport Science and University Sports, University of Vienna, Vienna, Austria; Western Michigan University, UNITED STATES

## Abstract

Basketball is a game of simultaneous actions, and inter-player coordination is key for offensive success. One of the most challenging aspects in this regard is basket cutting on a teammate’s drive. The ability to make these cuts is considered to be an artistic skill, mastered by only a handful of players. This skill is also hard to assess, as there is no method to measure the players’ capability with respect to this quality–especially not automatically. Using SportVU data from the NBA, we created a mathematical model that identifies the openings in the defense which allow to perform a cut. Our model succeeds to generalize, as it detects these openings on average 139ms earlier than the actual cuts start and has an overall (balanced) accuracy of 0.818 on the test set. Having a tree-based gradient boosting classifier, we received a clear hierarchy of feature importance and were able to inspect the interactions between these attributes during action. This way, the model gives insights about the kind of defensive movements needed for a player to allow enough space to cut while in practical usage the analysis of the output can also help the coaching staff in designing play options and assessing player abilities. By paying more attention to the possible off ball movements during drives, offensive plays can become more versatile–benefiting the participants and the spectators alike.

## Introduction

Many sport analytical models concentrate on event detection using e.g., Hidden Markov Models in basketball [1] or ensemble tree modeling in soccer [2]. Even the classification of more complex tactical elements was successfully implemented years ago [3,4], and the expansion of deep learning creates more insights about the value of the different actions happening on the field [5]. However, many of these methods–especially deep learning models trained with big data from past years–are biased towards the majority class [6] and thus favor the traditional style of game play. Only limited research explores the alternative options that players *could have done* [7], or what choices would be expected in given situations [8].

As basketball is a fast-paced game–played only by five players per team–the coordination of all players is a crucial element of a successful offense [9]. Spacing and getting open is an important task for the players to create quality shots [10], but deciding how to perform these moves is complex [11]–especially if a dynamic action is taking place, such as a drive (which means attacking the basket off the dribble) by a teammate.

Due to the high emphasis on correct spacing, basket cutting (meaning a quick move to the basket in order to receive the ball and score) on drives became an underutilized option for creating easy shots. The ability to decide whether to cut–along with the right timing–is a well-respected skill mastered by only a small portion of players. Making a wrong cut in an inappropriate moment can shatter the spacing of the whole team. However, with proper execution, these cuts can be highly beneficial: They not only increase the variability of the offense (creating a positive effect on future plays), but generally end up under the basket (92.3% of the times in our dataset), in the area which has the highest expected points per attempt [12] and the best potential to draw shooting fouls. Bringing attention along with useful insights to the nature of these cuts can help experts utilize these possibilities more often–an exciting step to slow down the tendency of making plays mainly behind the three-point line.

Therefore, the purpose of this paper was to find the main characteristics of defensive movements that allow an offensive player to cut towards the basket. We modeled the formation of defensive gaps by using the mere positions of the players and trained an easy-to-interpret tree-based classifier to reveal the key attributes that are indicative for cut openings. We also explored the interactions between these attributes by analyzing the partial dependencies for single and double features [13], which gave a clearer picture about the relationship of directions and measure of defensive shifts needed to make a cut possible. Taken together, our analysis shows that mainly two key attributes of defender behavior are necessary to recognize cut possibilities. Coaches can use this information for teaching purposes, as well as for the assessment of their players’ actions.

Furthermore, the implementation of a model signaling such opportunities arising on the court can have great usage for analytical purposes: detecting these options gives coaching staffs a tool for recreating their play sets as much as assessing player and team performance. Additionally, we showed how the influence of the features can be analyzed by SHAP values for every single decision made by the model.

## Methods

### Data

We used the SportVU data set made publicly available on the internet [14] throughout our analyses. It includes time-sampled (25 frames per second) moment to moment high-resolution positional records from the first half of the 2015–2016 National Basketball Association (NBA) season. This data set is a reduced and raw version of the positional information that provides the source for the official tracking statistics and is also used for analytical research by the NBA teams [15]. Stephanos et al. give a detailed description about the importance and usage of SportVU data for game analysis [16]. Particularly this version has been used successfully in several studies, even for performance testing of deep network architectures [17].

Because this data set was a raw and unlabeled version, we implemented a system to identify the main actions on the court, such as dribbling, passing and shooting. The detailed extraction procedure of these event labels has been described by [18].

Note that in the used data set distances are given in feet. As the usage of this metric is widely spread in the field of basketball, we will also use it for court dimensions throughout this paper–with the SI equivalent given in parenthesis.

### Detecting drives and basket cuts

Defining an exact set of actions is of high priority when creating a model that generalizes well with the given conditions. For this reason, we filtered the possessions for halfcourt offense sequences. Fast break situations (meaning quick attempts right after gaining possession to move the ball upcourt and score–frequently by outnumbering the defense) have different characteristics, and our main interest was on finding the traits of a typical defensive rotation during drives against set defense (the filter for halfcourt offense sequences includes the minimum length of two seconds on the offensive halfcourt, and a restriction that the ball must come to a stop on the x-y axis at any point).

To find cuts that happen on the drive of a teammate, we first needed to detect drives and basket cuts:

1. The definition of drives can be found on the NBA website: *“When a player attacks the basket off the dribble in the halfcourt offense*. *Does not include situations where the player starts close to the basket*, *catches on the move*, *or immediately gets cut off on the perimeter”* [19]. For our purposes the catch on the move was not an excluding factor because our goal was to analyze the openings created by the fast movement of the ball handler to the basket, and not the origins of this act.
2. Finding a clear definition for a basket cut is more challenging, as neither the NBA nor the FIBA defines it on their official website. According to common basketball knowledge, it is a quick move to the basket with the purpose of receiving the ball and scoring. This notion is confirmed by a well-established basketball website: *“Dive Cut or Basket Cut*: *A dive cut is any cut toward the basket and will many times result in an easy lay-up for you or a teammate*.*”* [20].

As there were no labels or more exact descriptions available for drives and cuts–after careful analysis of over 30 offensive actions–we set thresholds for a rule-based detector in terms of three features:

- distance proportion: the reduction of the basket distance divided by the total distance covered since the last frame;
- speed: the total distance covered since the last frame divided by the time between the frames;
- basket distance: to exclude fast dribbling or sprinting far away from the basket, we also included the player’s distance from the basket into the rule set–making sure that the detected actions took place in the set offense and not too far away from the relevant areas.

The detection was based on these features, as we created the rules using the percentiles of their values: A frame had to be in the top 90% range of *speed* and *distance proportion* of the annotated drives and cuts in order to be marked with the respective action. Via this, we defined drives as movements with the ball with a speed over 5.23ft/s (1.59m/s) *and* a distance proportion of over 0.50; while cuts had to have a speed of at least 5.96ft/s (1.81m/s) *and* a distance proportion of over 0.77. Setting the thresholds for these two features, the two main characteristics that make up a drive and a cut were defined: the speed and the direction of the movement. Additionally, we set the minimum distance (for the first detected frame) from the basket to 8.5ft (2.59m) to exclude post moves, and the maximum distances were set to 28.4ft (8.66m) and 23.4ft (7.13m) respectively (0.9 percentile of the distances of the annotated drives and cuts). Because we were interested in basket cuts, we also added the rule that cuts had to end at the basket area (inside 8.5ft– 2.59m –distance from the basket) in order to be recognized.

Based on the annotation of additional 15 actions, the balanced accuracy of detection on frame level was close to 0.89 for both groups, as can be seen in Table 1. The starting moments of drives were on average 80.2ms late by the detector (std: 158.44ms), while in case of cuts the detector was 4.4ms late on average (std: 100.92ms).

**Table 1:** Results of the frame-based evaluation for detecting cuts and drives.

	CUTS	DRIVES
**Sensitivity**	0.786	0.789
**Specificity**	0.994	0.998
**Overall accuracy**	0.988	0.991
**Balanced accuracy**	0.89	0.893

### Creating features

**Fig 1:**
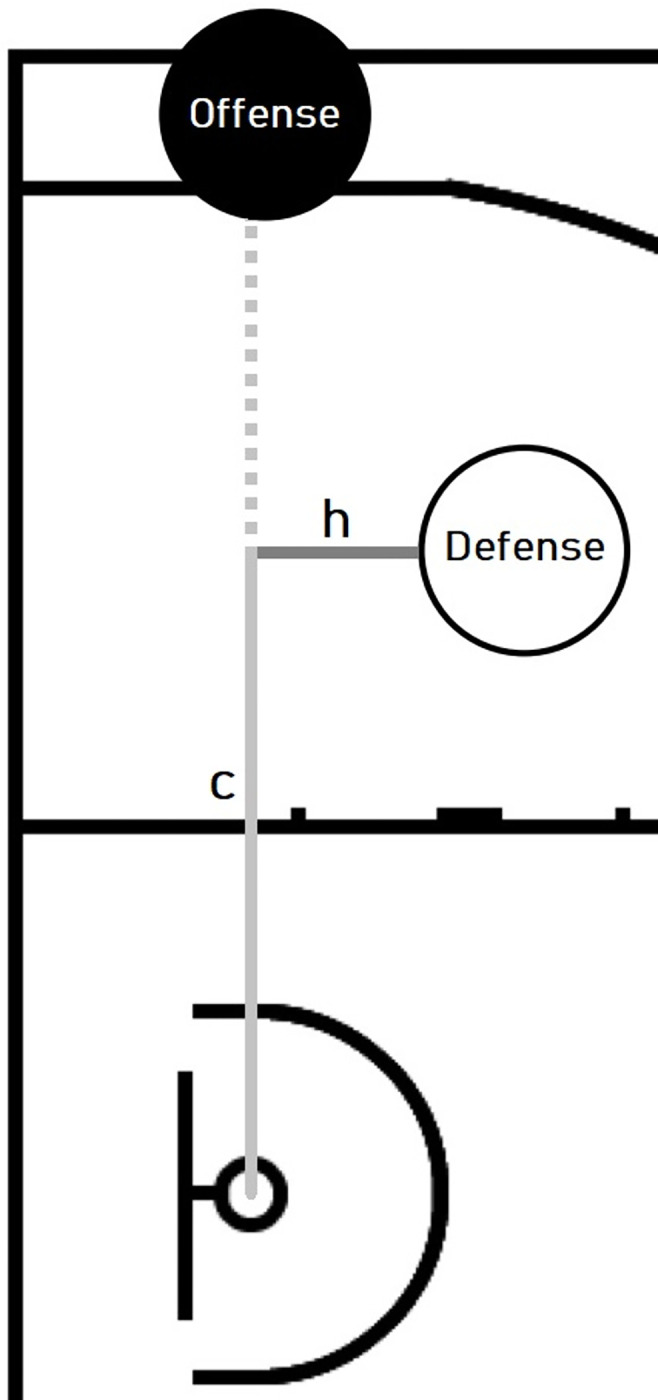
The vector components of the defender’s relative position to the offensive player and the basket–the two basic geometrical features h (distance between the defender and the direct path from the potential cutter to the basket) and c (distance between the projection point of the position of the defender onto the direct path of the potential cutter and the basket).

We used five main features as input for the model:

- *h*–Distance between the defender and the direct path from the potential cutter to the basket. (Fig 1: *h*–referring to the height of the triangle formed by the position of the two players and the basket)
- *c*–Length of the line starting from the projection of the position of the defender onto the direct path of the potential cutter and ending at the basket (Fig 1: *c*–the adjacent of the right triangle formed by the defender, the basket and the projection of the defender’s position onto the direct path of the offensive player to the basket).

From these two main features we derived three more attributes to detect movements from the last 200ms as well as a relative value:

- *h_change*: average change of *h* computed from the last five frames
- *c_change*: average change of *c* computed from the last five frames
- *c_relative*: *c* in proportion to the basket distance of the potential cutter

### Generating the training data

To select offensive actions that end with a successful cut on a drive, we filtered for halfcourt offenses in which the last action was a drive by the passer, and the receiver was in the act of cutting at the moment of ball reception (200 actions)–excluding the player who set the screen in a pick and roll (which is a different scenario because the screening action creates a gap for rolling to the basket very often). However, for the purpose of training and validating, the model needed actions where the decision was clearly correct, the cut was not overly curled in shape and the player was able to advance to the basket without having to navigate against defenders still in front. To account for this, we assessed the filtered sequences qualitatively, and chose 100 offensive actions that fulfilled these requirements. These actions were the basis of the positively labeled set for modeling.

Afterwards, we generated another set where the last action included a drive and kick for a catch and shoot attempt (262 randomly sampled actions). However, in these actions different technical / tactical reasons could also explain why a player chose to space out instead of cutting on the drive. Because of this, we selected qualitatively 100 possessions where the cut was apparently denied by a defender, and by that the decision to space out was not (only) due to technical or tactical reasons. These actions were the basis of the negatively labeled set for modeling.

As the classification of the model happened on frame level, it was vital to get the representative moments in these actions for the training. For the positively labeled cutting actions, we used 120ms prior and 120ms after the first moment of the marked cut. This way, the chances were very high that there was a detectable opening in the defense in these frames. As we didn’t have such a decisive moment for the negatively labeled drive and kick actions, we took the first twenty frames from the starting moment of the drive, ensuring that the relevant moments of cut denials were included. During the time of these frames, the driving player can reach the paint from the three-point line, providing the appropriate space for starting a cut off the ball (computed with the average driving speeds from the section *Detecting drives and basket cuts*–which is 10.45ft/s or 3.19m/s). This gave us an unbalanced number of frames but represented similar phases of the offensive sequence. The exceeding number of negatively labeled frames was favorable, as in basketball games spacing out for a spot up shot occurs significantly more often than cuts on the drive.

### Choosing the model type

As creating an easy-to-interpret model was one of our main purposes, we chose to train a tree-based model. This choice was well suited for the case of the features that interact with each other in a non-linear way. However, to overcome the problem of overfitting–for which decision trees are known–we looked for a boosting method to combine the outputs of many “weak” classifiers and produce a powerful “committee” [21]. With the scarce and complex tabular data–what such action sequences represent–gradient boosting seemed to bring the most fitting solution. The advantages stated by its inventor list every element we were looking for: “Gradient boosting of regression trees produces competitive, highly robust, interpretable procedures for both regression and classification, especially appropriate for mining less than clean data.” [13]. We used the Scikit-learn implementation accompanied by the default mean squared error with Friedman’s improvement score as criterion [22].

### Tuning and testing

To create the tabular input data with the same size, the training was carried out on the closest defender to the direct path between cutter and the basket (measured by the *h* value). This means that for the training procedure, only the primary defender was considered–however, on the action level we summed up the contributions of all defenders to account for every relevant obstacle for a cut.

To avoid overfitting, we used 5-fold cross-validation (each round 20 positive and 20 negative actions for testing) and trained the classifier on frame level with the 80 remaining actions. The training procedure started by generating the input data (as described above at the end of *Generating the training data* section), then dividing them into training (80%) and tuning (20%) sets. These tuning sets were used to optimize the learning rate parameter, which was set to 0.25 accordingly.

To test the performance, we ran the cross-validation models with the already chosen learning rate on the unseen group of test actions. The complete inference was done by summing up each defender’s probability results from the classification to account for the whole defense. The process included the assessment of two components: whether the system detected the outcome that was marked by the label, and in the case of true positives, the time of the first detection compared to the actual start of the cut mark.

### Using the model

To get a glimpse into the workings of the model, we also ran it on the remaining offensive actions (the ones that were not selected as clean enough for the training session) but filtered with slight restrictions: sequences got excluded that ended up with loss of possession, and cuts on the drive count only when the player receiving the ball ended inside 15ft (4.57m) distance from the basket. This rule was applied also in the reverse direction: Drive and kick actions count only if the receiver caught the ball outside this theoretical line of 15ft (4.57m) distance. This way we made sure that the actions represent successful basket cuts and at least mid-range distanced spot ups (88 and 117 respectively). Even though it was not a performance indicator of the model, we analyzed the overlap of player choices and model detections as well.

### Differences in player attributes

The goal of our model was showing the positional possibility of a cut during the drive of a teammate–independent of the personnel. This also means that great shooters that are intentionally spotting up as often as they can will have choices that are less often intact with the model output than ‘two-way’ players (in terms of shooting and finishing strong under the basket, which is often needed when a cut is performed). To analyze this effect, we looked at impactful attributes of the ball receiving players, such as height, weight and shooting ability. We created a statistic between both label sets (independent T-test) as well as calculated the statistical differences between the detection overlaps and distinctions (also independent T-test).

### Interpretation of the features and individual inferences

For determining the feature importance (also known as Gini importance), we used the Scikit-learn implementation [22] which computes the importance as the (normalized) total reduction of the criterion brought by that feature.

To interpret the interactions between the features, we included the most interesting partial dependence plots along with the analysis of an individual action as a case study and the SHapley Additive exPlanations (SHAP) values for the feature impacts at the first detection moment. SHAP values [23] are computationally tractable approximations of Shapley values, which show the average marginal contributions of feature values across all possible coalitions. To compute these values, we used the python implementation by [24].

## Results

### Model performance

We evaluated the model-performance on action level with cross-validation on the completely unseen actions in the test set. The model reached an average (balanced) accuracy of 0.818 with a standard deviation of 0.083. The mean of the sensitivity was 0.86 with a standard deviation of 0.074, while for specificity the mean was 0.78 with 0.135 standard deviation.

### Model reacts in time according to the detection / cut mark comparisons

To show that the model successfully generalizes to detect cutting possibilities, we also compared the times of the first detected openings and the starting times of the actual cuts in the true positive examples. We found that the model detected the openings 59.30% of the cases earlier than the player started performing the cut. These differences showed an average advance of 138.7ms for the model compared to the start marks.

**Fig 2:**
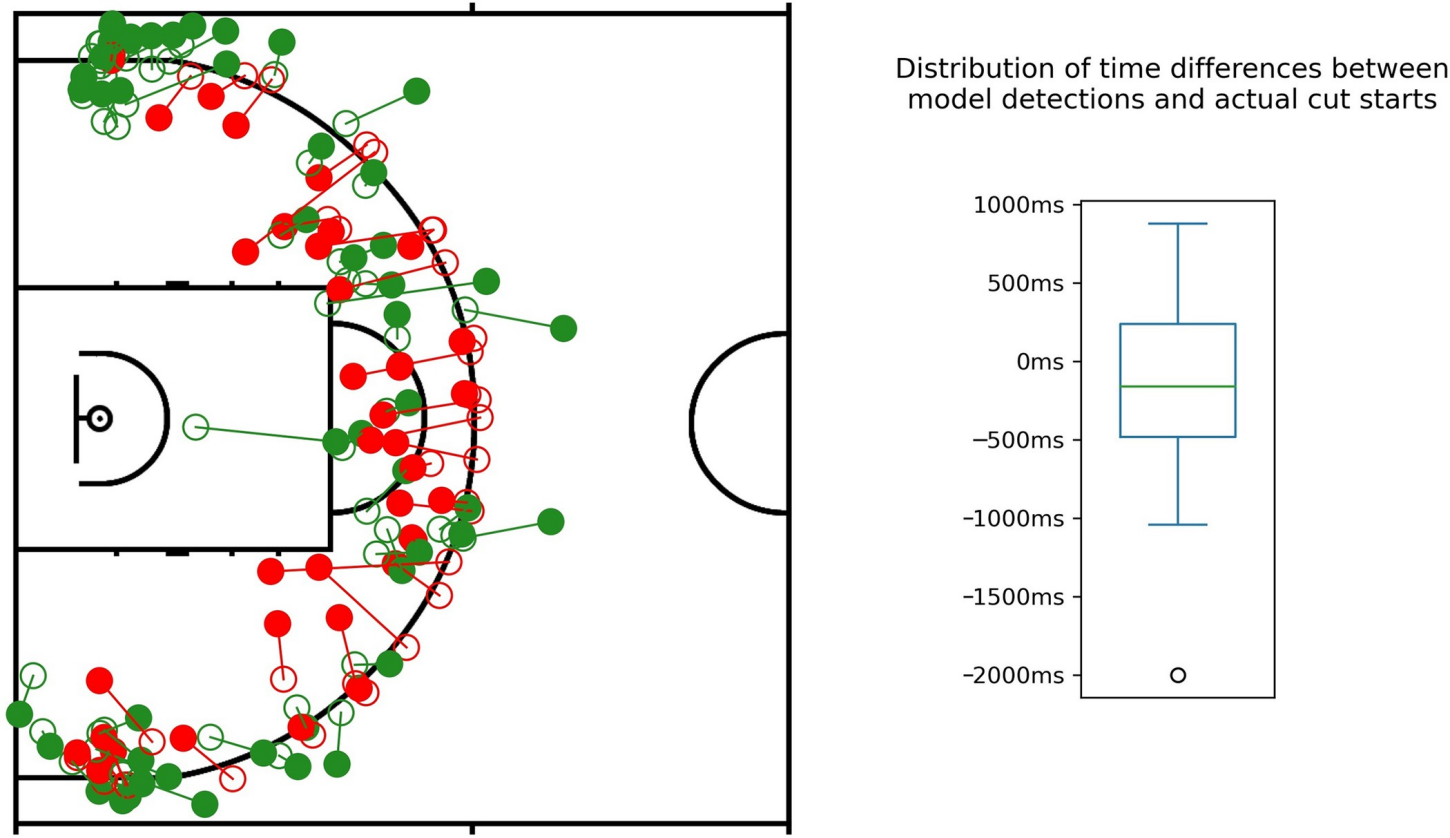
**Left side**—vector plot of early or equal (both green) and late (red) detections. The filled circle shows the player’s position at the moment of model detection of a cut opening, while the empty circles mark the position at the starting moment of the cut. **Right side**–Box plot of the distribution of time differences between first detected cut opening and actual cut start by the player.

By looking at the early / late detection cases on the court (Fig 2 –left side), we can confirm that the spatial distribution of early and late detections is close to being evenly distributed–we can only see a slight increase of early detections in the corners (especially on the top).

As the box plot on the right-hand side of Fig 2 reveals, the majority of the points are in negative territory, meaning that the detector was earlier than the start of the cut mark. We also analyzed the spatial distribution of these timings to get some insights why the model responded in different ways. The left side of Fig 2 shows how the early and late detections are spatially distributed.

For further analysis of the time differences in the quartiles of the box plot (Fig 2 –right side), we extracted the average feature values for the starting moments of the cut marks per group (Table 2)–having only one outlier at -2000ms. As we observed the examples, where the detections were ranging from being 480ms to 1040ms earlier (Q1), to the ones where the detection was 240ms to 880ms late compared to the actual cut mark (Q4), an increasing tendency for *c* and *c_relative* until they apparently reached a ceiling was found. At the same time *h* was decreasing rapidly over the whole range, while the two changing attributes (*h_change* and *c_change*) did not seem to have a strong relation to the quartile groups.

**Table 2:** Average feature values at the starting moment of the cut marks according to quartile groups.

	h (ft)	h_change (ft/40ms)	c (ft)	c_change (ft/40ms)	c_relative
**Q1**	5.03	0.03	7.26	-0.07	0.36
**Q2**	3.91	0.01	8.35	-0.19	0.41
**Q3**	3.74	0.1	11.05	-0.09	0.51
**Q4**	2.48	0.01	11.34	-0.1	0.5

### Depth of the defender position is more important than its closeness to the cutting path

**Fig 3:**
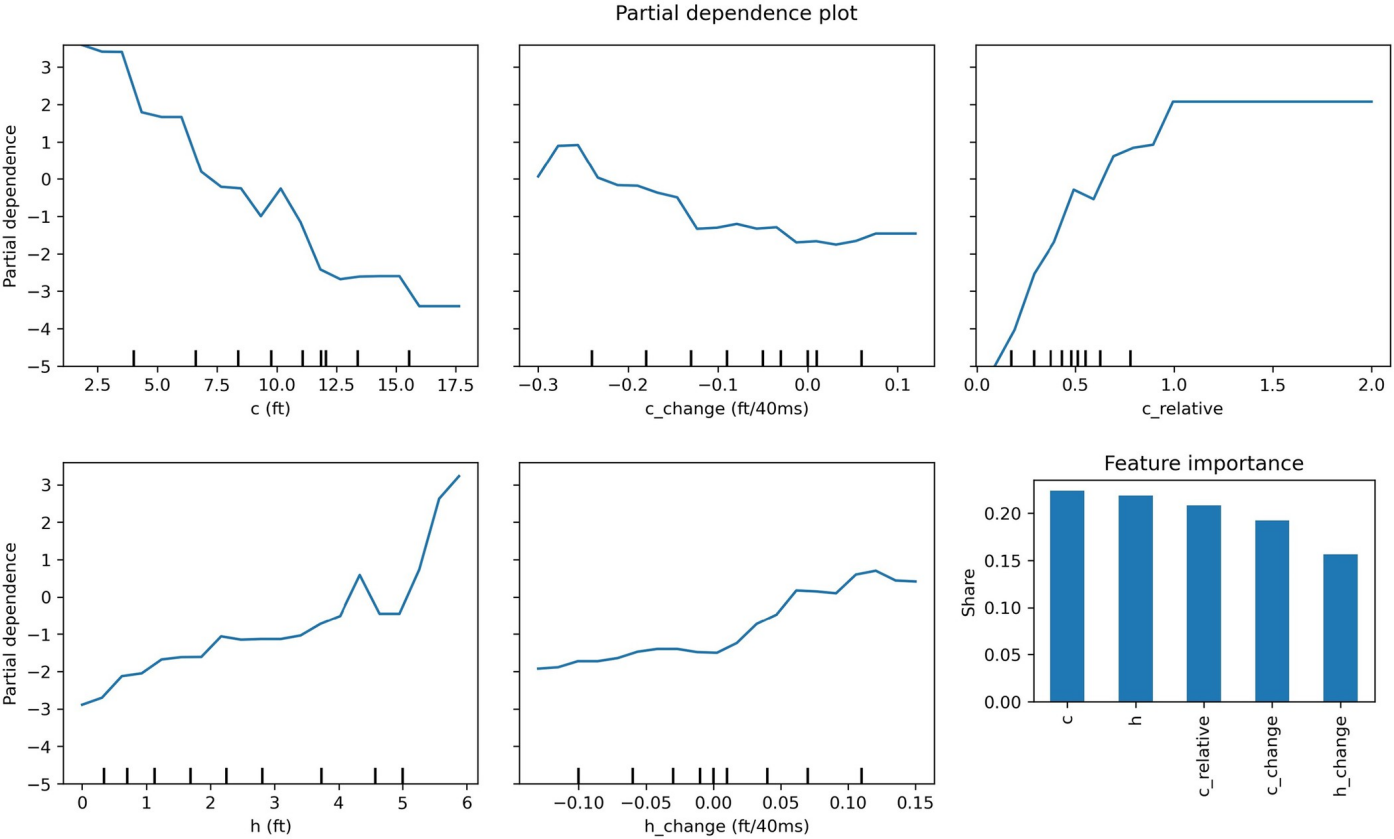
Single feature partial dependence plots (line plots) and feature importance according to the Gini index (bar plot—bottom right). Note that the y-axes in partial dependence plots are unitless.

We can see the partial dependence plots of single features in Fig 3 (line plots) showing the impact of the particular feature to the outcome–the higher on the y-axis, the more probable a cut was detected.

The bar plot (Fig 3 –bottom right) shows the overall interpretation of the feature importance according to the decrease in the Gini index [25]. Interestingly, the depth of the defender’s position (*c*) came first and proved to be even more important than how far the defender stayed from the direct path of the offensive player to the basket (*h*).

**Fig 4:**
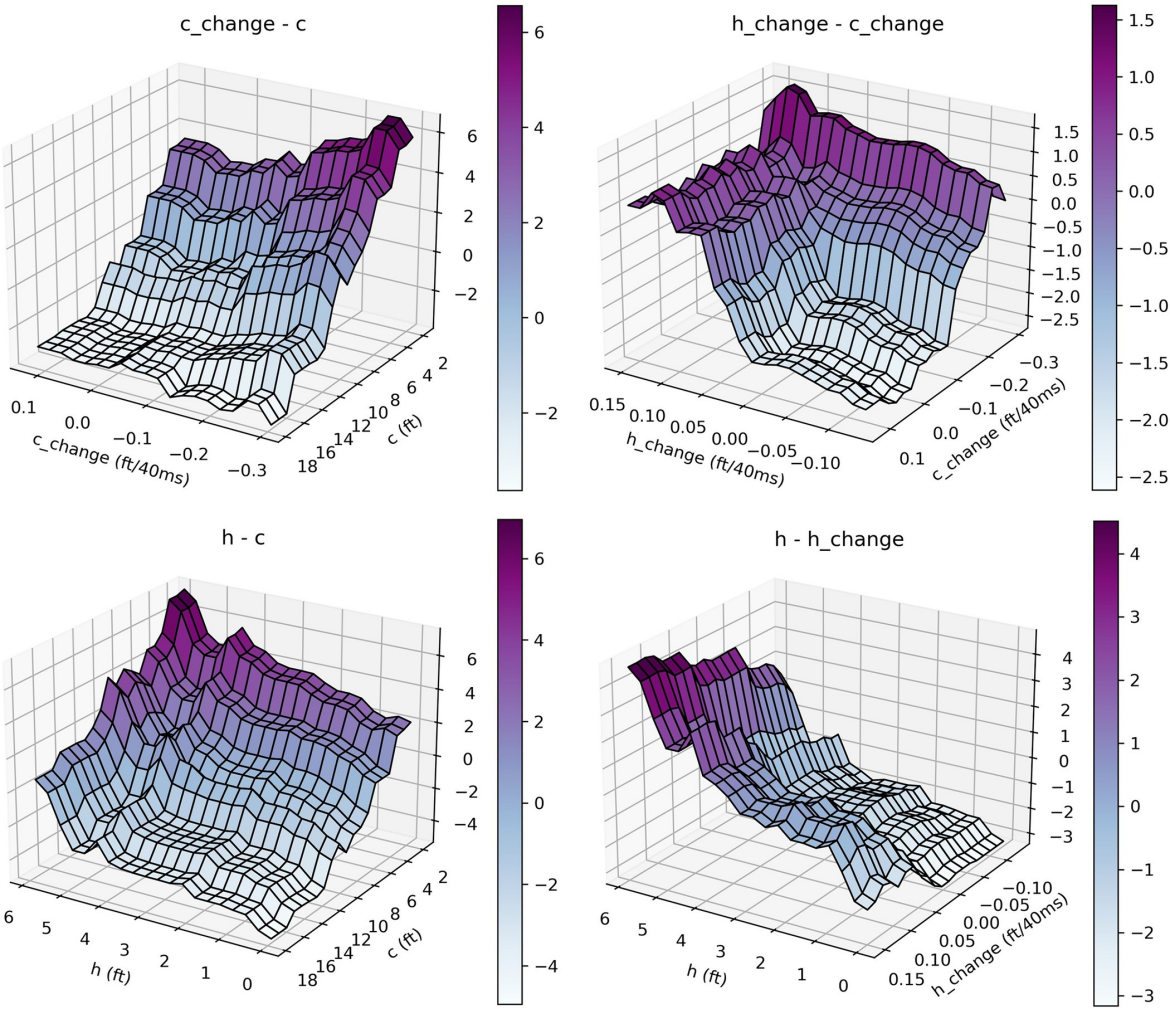
Two-feature partial dependence plots showing the interactions between the feature pairs. Note that the scaling is different–the impact of the feature-combos was not equally strong.

To investigate the interactions of the features, we also analyzed two-feature partial dependences, for which the plots can be seen in Fig 4. Even the interpretation of the quartile analysis (Table 2) becomes much more self-evident by looking at these partial dependence plots. As mentioned, *c* was increasing from Q1 to Q4 –and it also makes sense if we look at the single partial dependence plot of *c*–but the difference between Q3 and Q4 in this term was rather small as it seemed to have a plateau at around 11ft (3.35m). However, taking *h* also into account, we can track the impact of the two features combined on the two-feature partial dependence plot of *h* and *c* (Fig 4, *h—c*). The difference between the *c* values might not be relevant, but paired with the still lowering *h* values, the model was not able to predict the opening in time in case of the Q4 group. Taking a look at the *h—h_change* plot, we can confirm that the difference between Q3 and Q4 made an impact; an *h* value of 3ft (0.91m) gave a greatly different output with a value of *h_change* at around 0 than at 0.1ft/40ms (0.03m/40ms–we used this denominator for the unit because this time represents the difference between two consecutive frames). The scaling of these plots also reveals that the impact of the change features (*h_change* and *c_change*) alone was not highly relevant–a defender standing completely in the right position had to sink to the basket and detach from the offensive player’s path very quickly to create enough opening with other conditions being equal (though these two features worked together in quite a balanced way as we can see on the *h_change—c_change* plot). However, we can also see that both features had a clear threshold where they took off–a threshold probably meaning the commitment of the defender to helping on the drive. The plot of *c_change—c* confirms the notion that the change attributes worked hand in hand with their source feature. Without having the proper *c* value, *c_change* was not making a high impact. Note that the scaling is different on the plots–the impact of the feature-combos was not equally strong. The abrupt rise of *c* from around 4ft (1.22m) downwards can be explained through the fact that the restricted area stretches 4ft (1.22m) from the basket. In this area the defense has very limited options to stop a player coming at high speed towards the rim.

### Model reveals unutilized openings

To gain insights on how the model works in more chaotic situations, we ran it on the remaining filtered offensive actions, and compared the results with the actual choices of the players. The proportion of the overlaps was 0.72 with a sensitivity of 0.81 and a specificity of 0.63. Note that even though we use the same expressions for a better understanding, these numbers are no performance metrics, as many players made their choices due to technical / tactical reasons, as we will show next.

### Player choices depend strongly on their skills

Our model detects possibilities depending *only* on the positional situation. Analyzing the detection results, we can see that they reveal strong differences in player attributes. Such differences are obviously present between the cutting and spot up players in the differently labeled offensive actions (Table 3).

**Table 3:** Average attribute values of cutters and spot up shooters in the analyzed data set.

	cut	spot up	p value
**Height** (cm)	203.8	200.24	0.00000964
**Weight** (kg)	105.87	100.38	0.00000028
**3pt %**	27.75	31.9	0.00062781

For the detection comparisons, Table 4 shows how height, weight, and catch and shoot skills from behind the three-point line made a difference in model performance too–the model detects the openings, but the players chose another option, often out of different reasoning. The p values were computed between true positives and false positives as this pair of groups was best represented and most relevant.

**Table 4:** Average attribute values according to model detection results.

	true positives	false positives	true negatives	false negatives	p value
**Height** (cm)	204.56	200.96	199.78	203.37	0.041
**Weight** (kg)	106.65	99.91	99.71	104.39	0.005
**3pt %**	26.21	34.65	30.67	33.5	0.007

## Case study

**Fig 5:**
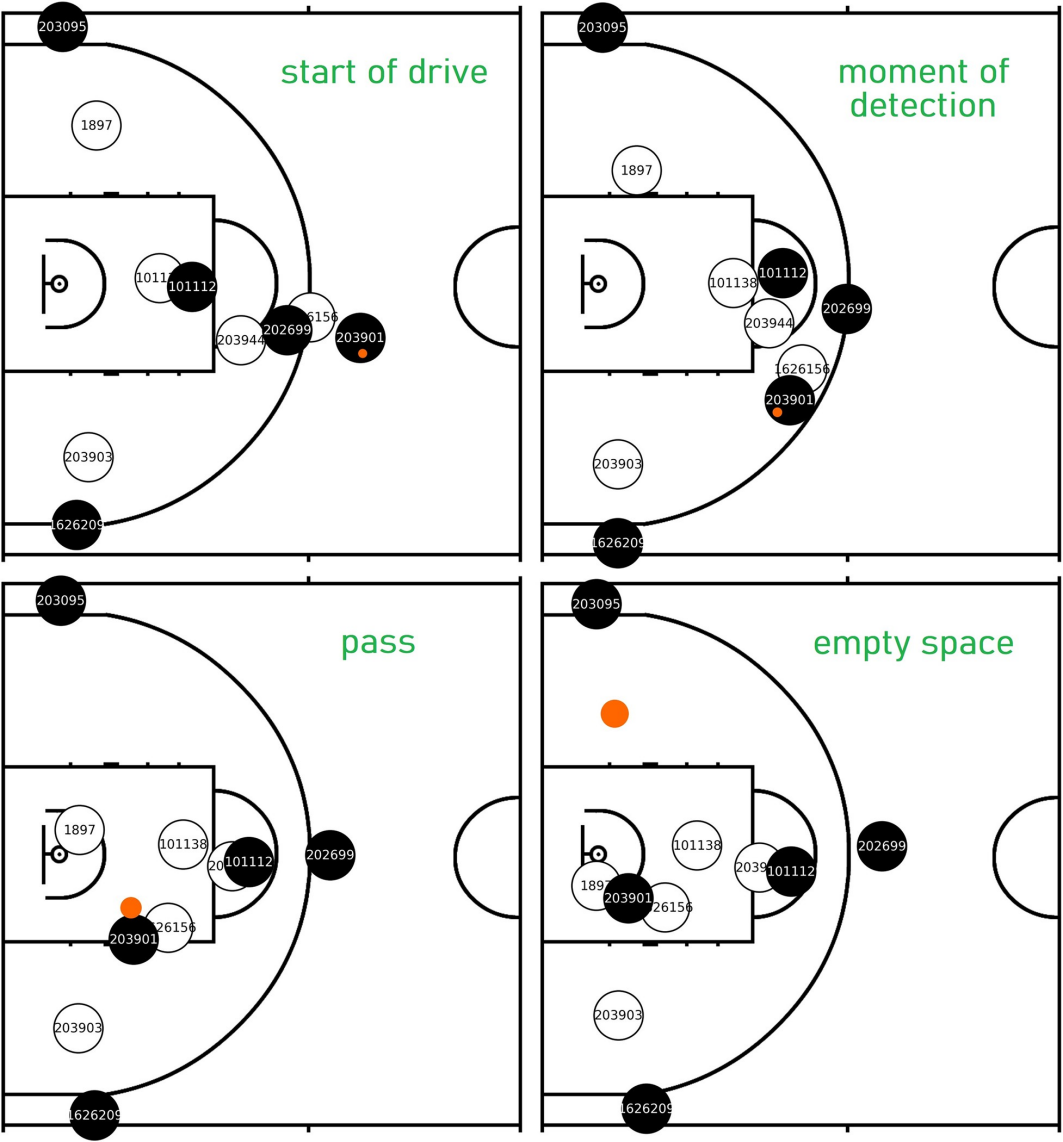
Example action for the detection of cutting opportunities. **1)** Drive starts off the ball screen; **2**) the moment of detection during the drive—the defender is hasting to rotate for helping; **3**) at the moment of the pass the whole right (upper) side of the court is free from defenders; **4**) The unfolding of the action–the right side of the court is still an empty space, although the pass already left the paint area.

Fig 5 shows an action from the unselected data set. The model detected the possibility of a cut well before the driving player was in the act of passing. The sequence of moments clearly confirm that the defensive rotation created enough space for a cut, but Evan Fournier (203095) chose to stay on the perimeter instead (he shot this season 41.4% from behind the three-point line out of the catch, which makes his decision easily comprehensible).

Most notably, the value of the feature *c* was sinking quickly, which brought a relatively high absolute value of *c_change* with -0.30ft/40ms (-0.09m/40ms). However, none of these features alone could cause a positive output for the detection moment; as we saw on Fig 3, a value of 10.69ft (3.26m) for *c* had still a negative impact, while -0.30ft/40ms (-0.09m/40ms) for *c_change* alone was also on the border. Their combination made already a strong positive effect (Fig 4 –top-left), especially considered that the defender had almost no movement towards the cutting path by *h_change* being -0.01ft/40ms (-0.03m/40ms), which was also a positive pairing with this value of *c_change* (Fig 4 –top-right).

**Fig 6:**
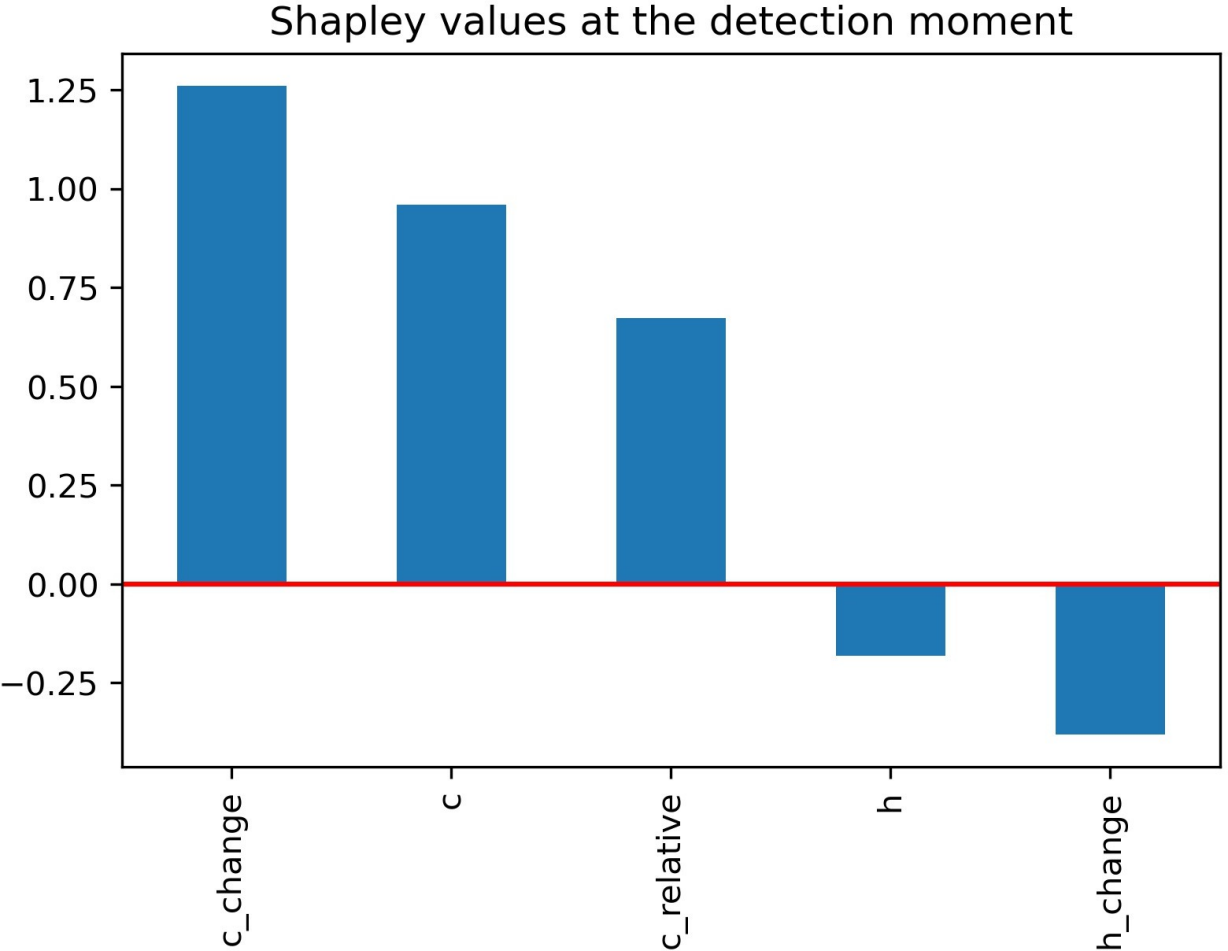
SHAP values of the features derived from Metta Sandiford-Artest’s movement at the moment of first detection of an opening in the case study example.

The SHAP values were close to the partial dependence analysis: Fig 6 shows that *c_change* had the largest impact on this particular (positive) detection, followed by the rest of the *c*-dependent features. The *h* value at this moment was simply too small to have a positive effect and as *h_change* was also negative–even if only a small amount–it was a movement by the defender toward the cutter’s path and therefore had even a slight negative impact for an opening.

Described with less technical terms, in this situation Metta Sandiford-Artest (1897)–the defender–committed to helping on the drive and would not have been able to cover the cut. He moved towards the basket quickly (meaning a decrease in *c* and bringing *c_change* deep in the minus territory) because he anticipated that there was no one else to prevent an easy layup. He wanted to meet the ball handler at the rim, so he did not move higher towards the free throw line (causing no increase in *h*)–rather neared the baseline. The model recognized this intention very early based on the vector components of his movement. There would be no other player in position to help the helper either (further away Brandon Bass– 101138 –was dealing with the drive and protected a possible roll of his man until the pass occurred), so the model only had to account for the primary defender in this situation for generating the output.

## Discussion

While the detection and evaluation of events have a huge impact on sports–e.g., by driving changes in shooting policies of soccer teams [26], discovering unutilized possibilities is a less frequent topic in sport analytics. We trained a classifier to recognize cutting opportunities during driving actions–a play option that gets arguably less attention than its potential.

As the number of three pointers per game are wildly rising [27], teams are built offensively to make these shots efficiently, and defenses start to adjust their game plans–e.g., [28] weighs on how ‘staying home’ and helping on the drive options could be distributed for defending the corner spot-up shooters using game theoretical analysis. However, the offense can still make the plays more unpredictable; cutting on the drive is such an option for players away from the ball. As [29] described, inexperienced players dribble and cut more often than players on higher levels. This finding makes the topic even more pressing, as wrongly timed cuts have the potential to exceedingly harm the efficiency of the offense on the professional level. Yet, with careful attention to details, cutting is an extremely beneficial skill even for good shooters to mix up their game.

By paying more attention to these situations, the entropy of the offensive plays can be increased which is arguably elevating the expected points. While D’Amour et al. showed the positive effect of higher entropy for the offense by moving the ball [30], a logical implication would be that this result also transfers to the concept of off-ball behavior during a drive–especially because the defense gives different responses as well. Furthermore, less skilled shooters can be more involved if they are capable of making a well-timed cut. Cutting players in our data set tended to be generally much less effective from the three-point line (27.75%) than their counterparts who chose to spot up in these analyzed actions (31.90%). The difference in expected points between the two groups of actions (1.255 for the cuts vs 1.030 for drive and kicks) also implies that the potential of cutting on drives is far from being exhausted (p = 0.040).

To get closer to this potential, the purpose of our model was finding opportunities where a player could benefit from the defensive rotation forced by the drive and make a cut to the basket. By modeling the main attributes of the defense from the potential cutter’s standpoint, we created a classification system that can signal these openings. To obtain easy interpretability, we chose a tree-based algorithm with gradient boosting. Despite the booming of deep networks, this type of modeling is still highly performant [31], on one hand because its need for training data is low, and because of its more intuitive transparency. We obtained a reasonably good performance respective to the complexity of these situations.

As the classification of the model is frame-based, we were able to analyze the exact timings between the first detected cut-opening moments and the starting moments of the actual cut marks for the true positive cases (Fig 2, right side & Table 2). The comparison showed that the model was often signaling the openings earlier than the player started to act. It does not necessarily mean that the player was slower in recognizing the situation, since we don’t know whether they were ready to make an immediate cut given their physical settings. We also analyzed whether there was spatial correlation of the time differences between detected and performed cuts (Fig 2, left side). We found a slight increase of early detections in the corners, which can be explained by the fact that the middle is more jammed by defenders and the model had a harder time to detect openings–even if it is accounting for all participants, dealing with multiple defenders seems to be more complex and perhaps by this also more error prone.

One extra advantage of the frame-based approach could be that it enables the system to work on the fly–which has the potential to bring some benefits in live action; theoretically it could give real time feedback to players on the court during practice.

The direction and amplitude of the defender’s movement tell a lot about the upcoming gaps in the defense. Our analysis shows that by carefully observing two key attributes of defender behavior it is possible to recognize cut possibilities. From a practical perspective, we identified two scenarios that frequently lead to cut possibilities that can be focused on in training sessions. First, we found that the depth of the defender (*c*) was more important for the model output than the defender’s distance from the direct path between the offensive player and the basket (*h*)–and *c_change* also proved to correlate stronger to cutting possibilities than *h_change*. We argue that this difference is due to the fact that defenders are not detaching from this direct path without any reason, and in case of helping or rotating to help a helper the movement towards the basket is usually more prominent. Second, a high *h* value is an indicator for situations where a cut is unguardable almost regardless of the other features. It occurs usually in situations where a player in the defense loses his/her orientation and will occur more frequently in an amateur league.

As opposed to some prevalent way of thinking, defenders break the ‘triangle rule’ less often than cuts become possible. In this rule, the triangle is formed by the particular defender, the offensive player assigned and the ball handler. If it gets closer to fitting onto a line, the defender cannot see both the ball and his/her assignment, meaning that one of them will be lost sight of. While observing the triangle is a wise advice, it is also hard to assess during the game. Moreover, a meaningful deformation of this triangle turns out to happen less often than the above-mentioned shifts of movement components. The question whether the player and the situation fully meet the criterion for a successful cut is often more complex (e.g., NBA players often need little space for making a dunk or even an alley-oop, while other leagues have less athletic players) and hard to measure. However, the topic is well worth the effort because the easy shooting opportunities, the potential free throws and the long-term unpredictability of the offense have a huge potential payoff for a team of any level. Besides the general insights derived from the hierarchy of the features, model interpretation can also be benefitted in the case of individual actions, as SHAP values display the impact of all features for single detections (see our case study).

Despite scarce data, our model was able to perform well on the test set and detected cuts with a high overlap rate when probed on less ‘clean’ situations. The lower sensitivity in this category is indeed highly welcomed, as the purpose of the model is to identify cutting possibilities–even if the player did not choose to perform it. As these situations were not manually selected, the model had a good chance to signal situations where the player could have cut, as such sequences also contained actions where the players presumably made their choice depending on factors other than the positional setup (e.g., technical skills or tactical duties). To make it more apprehensible, we analyzed the differences of player attributes between the cutter and the spacer populations. Unsurprisingly, better shooters space out and spot up more willingly than more robust and taller players, who are more prone to take the cut when the moment arises.

The model can be improved in several ways; an exact use-case could include restrictions to the detection of cutting opportunities and could be easily implemented by a rule-based system. At this moment, the model infers based on the training data only–even though there are clearly defined patterns that could be added as a restriction to decrease the number of false positives: drives from the baseline open the lane at around 45 degrees on the opposite side for cutting and vice versa; middle drives make it possible to cut on both baselines depending on the exact situation. Most coaches will also have scenarios where they explicitly do not want any of their players to cut–these situations could also be avoided by the detector when it is fed by such rules. Although these additions are easy to implement, there is a need for more data to integrate them into the training iteration.

Superior players can also perceive telling signs about their defender not paying attention–i.e., turning their faces away for a moment–and in extreme cases it can also mean an opening for a cut. Obviously, the inability to recognize such possibilities is a limitation of our model, as the input data contains only the court positions and no postural information. It is not the most common case for such cuts–as we could see in the analysis–however with the advance of posture recognition [32] even this aspect could be considered. Another limitation of our analysis is that we can only compare the model timings to the detected cut markings based on positional information alone–meaning that we don’t know when the player starts to think about the possibility of cutting, and how much it impacts their movement when they are eventually preparing for it. Nevertheless, our model succeeds to generalize based on its positional input and provides valuable feedback about the cutting mechanism during drives.

## Conclusion

We modeled a complex recognition skill with tree-based gradient boosting using five features corresponding to the defenders. The feature analysis and the practical usage of the model can help coaching staffs and players to bring more attention to their cutting game and give hints to the factors that should be considered and trained for its success. The movement of the defender gives insights, if an opening in the defense is to be expected–and assessing the skill of recognizing it can bring some light on why some teams are superior in their cutting game.

## Supporting information

S1 Table Drive attributes. Provides the data considered during the drive detections (Methods/Detecting drives and basket cuts).
(CSV)

S2 Table Cut attributes. Provides the data considered during the cut detections (Methods/Detecting drives and basket cuts).
(CSV)

S3 Table Drive annotation times. Provides the results of the evaluation of drive detections (Methods/Detecting drives and basket cuts).
(CSV)

S4 Table Cut annotation times. Provides the results of the evaluation of cut detections (Methods/Detecting drives and basket cuts).
(CSV)

S5 Table Detection performance in absolute numbers. Shows the performance of the drive and cut detection in absolute numbers (Table 1).
(CSV)

S6 Table Spot-up sequences list. Lists the randomly selected drive and kick sequences, alongside with the information whether they made the qualitative screening for training and whether they got used when we run the model to see its workings (Methods/Generating the training data, Methods/Using the model).
(CSV)

S7 Table Cut sequences list. Lists the randomly selected cut on drive sequences, alongside with the information whether they made the qualitative screening for training and whether they got used when we run the model to see its workings (Methods/Generating the training data, Methods/Using the model).
(CSV)

S8 Table Frame scores. Displays the models’ performances for tuning the learning parameter (Methods/Tuning and testing).
(CSV)

S9 Table Model performance. Displays the model’s performance during the cross-validation testing (Results/Model performance).
(CSV)

S10 Table Timing differences. Displays the first time of detection/cut marks for the true positive cases (Fig 2 - right side).
(CSV)

S11 Table Q values. Displays the values of the features for the members of the quartile groups (Table 2).
(CSV)

S12 Table Positional Qs. Contains the positions of the members of the quartile groups (Fig 2 - left side).
(CSV)

S13 Table Single-feature partial dependence: c. Contains the numbers for the single feature partial dependence plots (Fig 3).
(CSV)

S14 Table Single-feature partial dependence: c_change. Contains the numbers for the single feature partial dependence plots (Fig 3).
(CSV)

S15 Table Single-feature partial dependence: c_relative. Contains the numbers for the single feature partial dependence plots (Fig 3).
(CSV)

S16 Table Single-feature partial dependence: h. Contains the numbers for the single feature partial dependence plots (Fig 3).
(CSV)

S17 Table Single-feature partial dependence: h_change. Contains the numbers for the single feature partial dependence plots (Fig 3).
(CSV)

S18 Table Feature importance. Contains the numbers of the feature importance plot (Fig 3 - bottom right).
(CSV)

S19 Table Two-feature partial dependence: c_change-c. Contains the matrix for the two-feature partial dependence plots (Fig 4).
(CSV)

S20 Table Two-feature partial dependence: h_change-c_change. Contains the matrix for the two-feature partial dependence plots (Fig 4).
(CSV)

S21 Table Two-feature partial dependence: h-c. Contains the matrix for the two-feature partial dependence plots (Fig 4).
(CSV)

S22 Table Two-feature partial dependence: h-h_change. Contains the matrix for the two-feature partial dependence plots (Fig 4).
(CSV)

S23 Table Cutting player attributes. Contains the attributes for cutting players in the cutting sequences (Table 3).
(CSV)

S24 Table Spot up player attributes. Contains the attributes for spot up players in the drive and kick sequences (Table 3).
(CSV)

S25 Table True positive detection player attributes. Contains the attributes for players in the true positively detected sequences (Table 4).
(CSV)

S26 Table False positive detection player attributes. Contains the attributes for players in the false positively detected sequences (Table 4).
(CSV)

S27 Table True negative detection player attributes. Contains the attributes for players in the true negatively detected sequences (Table 4).
(CSV)

S28 Table False negative detection player attributes. Contains the attributes for players in the false negatively detected sequences (Table 4).
(CSV)

S29 Table SHAP values. Contains the numbers of the SHAP values plot (Fig 6).
(CSV)

S30 Table Expected points per category. Contains the series for calculating the expected points for cuts and drive & kick actions (Discussion).
(CSV)

S1 File Sequence annotations. Contains action/event labels for the 462 analyzed sequences used in the paper. With the help of the gameIDs and times (last 8 digits of the real time entries), it can be bound to the raw SportVU data available online: https://github.com/linouk23/NBA-Player-Movements/tree/master/data/2016.NBA.Raw.SportVU.Game.Logs.
(ZIP)
